# A retrospective analysis of the etiologic agents and antibiotic susceptibility pattern of uropathogens isolated in the Jigme Dorji Wangchuck National Referral Hospital, Thimphu, Bhutan

**DOI:** 10.1186/s13104-015-1728-1

**Published:** 2016-01-29

**Authors:** M. Adeep, T. Nima, W. Kezang, T. Tshokey

**Affiliations:** Microbiology unit, Department of Laboratory Services, JDWNRH, Thimphu, Bhutan

**Keywords:** Urinary tract infection, *Escherichia coli*, Jigme Dorji Wangchuck National Referral Hospital (JDW/NRH), Etiologic agents, Uropathogens, Inpatient and outpatients

## Abstract

**Background:**

Urinary tract infection is one of the major public health problems. Specific population studies to understand the common etiologic agents and antibiotic susceptibility patterns are important to determine the empirical treatment of urinary tract infections. This is the first study in Bhutan to analyze the etiologic agents and antibiotic susceptibility pattern of uropathogens isolated from patients visiting Jigme Dorji Wangchuck National Referral Hospital with the ultimate goal of guiding empirical treatment.

**Methods:**

Hospital based (inpatients/outpatients) retrospective cross sectional study of 6030 clinically suspected patients with urinary tract infections who have submitted urine samples for culture in a 6 months period was done. Urine samples were collected and processed as per standard microbiological procedures and antibiotic susceptibility testing performed by CLSI guidelines.

**Results:**

Significant bacteriuria were detected in 14.9 % of the total patients. The most common uropathogens isolated were *Escherichia coli* (79.3 %) followed by *Klebsiella pneumoniae*. Females around the age group of 18–26 have the highest prevalence of urinary tract infection. The highest rate of antibiotic resistance was seen in amoxicillin (71.4 %) and nalidixic acid (80.3 %), and resistance were lower in nitrofuration (3.4 %) and gentamycin (17.5 %). The third generation cephalosporin resistance (which is a surrogate marker of ESBL) was 16.1 % in outpatient and 16.7 % approximately in inpatient setting.

**Conclusion:**

*Escherichia coli* was the predominant uropathogen making up 79.3 % (outpatient 81.1 % and inpatient 69.5 %) of the total and its antibiotic susceptibility pattern needs to be considered for treating community-acquired UTIs empirically. The third generation cephalosporin resistance (which is a surrogate marker of ESBL) is alarmingly high among the isolates and there is need for further studies.

## Background

Urinary tract infection is considered to be a major public health problem worldwide with an estimate of 150 million cases recorded per year [1]. The management of UTI has been profoundly easy with the introduction of antimicrobial susceptibility testing [2]: however antimicrobial resistance is a growing problem and is a major concern in many countries [3]. There is no study on this subject in Bhutan till date. Many studies have proven *Escherichia coli* to be the commonest causative agent of UTI [3–6]. However other agents like other enteriobacteriaciae, *Acinetobacter* spp. and Gram positive organism such as *Staphylococcus saprophyticus,* other *Staphylococcus aureus* and *Enterococcus* spp. are also commonly involved [3, 4, 6].

The resistance to many of the commonly prescribed antibiotics for UTI like amoxicillin, co-trimoxazole and norfloxacin has emerged over the past decade [7–10]. In our laboratory too, we see lot of resistant bacteria in our daily work but this has never been formally studied. Since most of the UTI are treated empirically, the selection of the antimicrobials agents should be determined by the most likely pathogen and its resistance pattern in a particular community [11–13]. Therefore the best choice of empirical treatment should be determined and there is a need of periodic monitoring of etiologic agents of UTI and their resistance pattern [12, 13].

## Methods

### Study design

This was a retrospective analysis study. The study was carried out in the microbiology unit of the Department of Laboratory Services, Jigme Dorji Wangchuck National Referral Hospital. The 350 bedded hospital, caters to patients from all over the country being the country’s national referral hospital.

### Study population and study duration

The study was a retrospective review of the urine cultures done in the microbiology unit of the Department of Laboratory Services for 6 months from January to June 2013. It included all urine samples received during the defined period from all types of patients. It involved only review of the sample processing registers for that period. From the register inpatient and outpatient samples were identified. Though it was very difficult to completely ascertain that all inpatient samples are hospital acquired UTI, for the estimate of UTI acquired in community and hospital, all the outpatient samples have been treated as community acquired-UTI. In addition since the register doesn’t have the details of clinical syndrome we assume that all the outpatient samples are from simple/uncomplicated symptomatic cystitis as without the symptoms patients are not sent for urine culture.

Bacterial isolates were identified by routine procedures in the laboratory and the antibiotic susceptibility testing was done by the CLSI method [2].

### Data collection

No patient details that may link to the patient identity like names was used and the confidentially was maintained. Data was collected from the register which included only demographic features like age, sex, location (outpatient or inpatient) and antibiotic susceptibility results.

### Data analysis

Data were entered and analyzed using SPSS version 14. Discrete variables were expressed as percentages and proportions were compared using the Chi square (χ^2^) test. Statistical difference were considered significant at p value of <0.05.

### Ethical clearance

Permission for waiver of ethical clearance was provided by Research Ethics Board of Health (REBH), Ministry of Health, Bhutan, since this is just a retrospective analysis of the urine culture results from patient registers. No patient details were used for analysis.

### Sample processing

Antibiotic susceptibility testing was performed for the isolates as per the CLSI guidelines [2]. The primary isolates were inoculated into Mueller–Hinton agar plates with antibiotic disks and incubated for 18–24 h at 37 °C. The following antibiotic disks were used: amoxicillin 10 mcg, cefotaxime 30 mcg, ceftriaxone 30 mcg, gentamicin 10 mcg, nitrofurantoin 300 mcg, cefazolin 30 mcg, ceftriaxone 30 mcg, nalidixic acid 30 mcg, norfloxacin 10 mcg, co-trimoxazole 25 mcg, gentamicin 10 mcg, nitrofurantoin 300 mcg, cefoxitin 30 mcg, vancomycin 30 mcg, penicillin G 10units, erythromycin 15 mcg. The results break points were interpreted according to Clinical and Laboratory Standards Institute guidelines [2]. ATCC strains for quality control used are *E. coli* ATCC 25922, *Pseudomonas aeruginosa* ATCC 27853 for Gram negatives and *S. aureus* ATCC 25923 for Gram positive isolates.

## Limitations

1. Because of the limited patient details entered in the register it was impossible to provide clinical and previous therapeutic details including the catheterized patients.
2. Though the register indicates inpatients and outpatients, ascertaining definite hospital acquired UTI among inpatients was difficult.
3. Though CLSI method was established and practiced for all microbiological processing, there may have been some discrepancies depending on the sample collection and processing performed by different staff on duties at different times.

## Results

A total of 6030 urine samples from clinically suspected patients were tested and analyzed for the study comprising of 1156 male and 4874 female patients over a period of 6 months. The age range of the patients was between 18 days and 87 years with a median of 40.1. A total of 863 (14.3 %) samples were culture positive showing significant bacteriuria and the remaining 5167 (85.6 %) were either insignificant or with sterile urine. Outpatient had a prevalence of 14.8 % and inpatient had 12.1 % respectively.

Out of 6030 patients in total, 4874 (80.8 %) were female and among these 729 (14.9 %) showed culture positive significant bacteriuria. From 1156 male patients, only 125 (10.8 %) had UTI. Female gender was a significant risk factor for acquiring UTI (OR 1.44, 95 % CI 1.17–1.76, and statistically significant *p* value of <0.001. The incidence was found to be more in female especially in the age group of 18–26 than the other counter parts of all age groups.

From a total of 863 isolates *E. coli* alone accounted 685 (79.3 %), other Gram negative isolates 104 (12 %), Gram positives 53 (6.1 %) and 9 (1 %) *Candida* spp. *E. coli* were the most pre-dominant isolate with the frequency of 81.1 % among the outpatient and 69.5 % in outpatient setting, causing UTI followed by *K. pneumoniae* 36 (4.1 %). Table 1 illustrates the overall frequency of uropathogens isolated.

**Table 1:** Frequency of uropathogen isolates

Isolates	Outpatient		Inpatient		Overall prevalence (no, %)
	No	%	No	%	
*E. coli*	594	81.1	91	69.5	685 (79.23)
*K. pneumonia*	30	4.1	6	4.6	36 (4.1)
*Enterococcus* spp.	14	1.9	9	6.9	23 (2.6)
*S. saprophyticus*	16	2.1	4	3.1	20 (2.3)
*Acinetobacter* spp.	14	1.9	4	3.1	18 (2.08)
*P. mirabilis*	12	1.6	2	1.5	14 (1.6)
*Pseudomonas* spp.	9	1.2	3	2.3	12 (1.4)
*K. oxytoca*	7	0.9	3	2.3	10 (1.1)
*Citrobacter*	8	1.1	1	0.8	9 (1)
*P. vulgaris*	7	0.9	2	1.5	9 (1)
*S. aureus*	6	0.8	1	0.8	7 (0.8)
*Enterobacter* spp.	7	0.9	1	0.8	8 (0.9)
*Streptococcus* spp.	2	0.3	1	0.8	3 (0.3)
*Candida* spp.	6	0.2	3	2.3	9 (1)

**Fig. 1:**
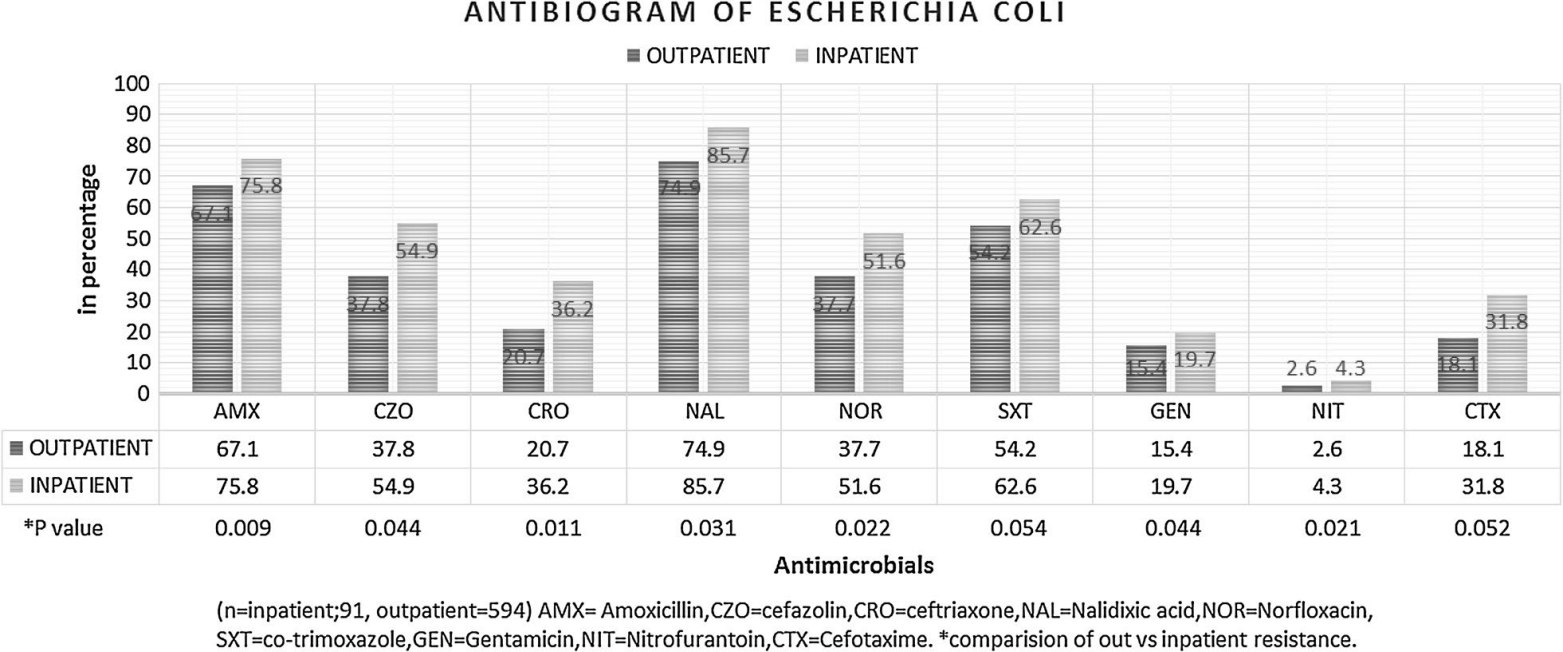
Antibiogram of *E. coli*

A total of 12 different antimicrobials were tested against all uropathogens, among which nitrofurantoin, gentamicin, ceftriaxone and cefotaxime were most effective than other antimicrobials such as: amoxycillin, nalidixic acid and co-trimoxazole. The percentage of antimicrobial resistance of the different uropathogen isolates to a panel of antimicrobials routinely used is summarized in Table 2. Overall Gram negative isolates showed higher resistant pattern in comparison with Gram positive isolates. *Escherichia coli* being the predominant cause of UTI infection showed highest resistance to commonly used antimicrobials: nalidixic acid (Out 74.9, In 85.1) followed by amoxycillin (Out 67.1, In 75.8) and co-trimoxazole (Out 54.2, In 62.6). Least resistance was seen in nitrofurantoin (Out 2.6, In 4.3), followed by gentamicin (Out 15.4, In 19.7). Figure 1 illustrates.

**Table 2:** Resistance rate of different isolates from inpatient and outpatient settings

Isolates	Antibiotics																							
	AMX		CZO		CRO		NAL		NOR		SXT		GEN		NIT		CTX		VAN		PEN		ERY	
	IN	OUT	IN	OUT	IN	OUT	IN	OUT	IN	OUT	IN	OUT	IN	OUT	IN	OUT	IN	OUT	IN	OUT	IN	OUT	IN	OUT
*E. coli* (n* = 91/594)	75.8	67.1	54.9	37.8	36.2	20.7	85.7	74.9	51.6	37.7	62.6	54.2	19.7	15.4	4.3	2.6	31.8	18.1	–	–	–	–	–	–
*K. pneumonia* (n* = 6/30)	–	–	66.6	30	66.6	10	33.3	20	16.6	13.3	50	26.6	50	3.3	16.6	10	50	10	–	–	–	–	–	–
*Enterococcus* spp. (n* = 9/14)	55.5	14.2	–	–	–	–	–	–	33.3	50	0	14.2	–	–	0	7.1	–	–	0	0	100	57.1	88.8	57.1
*S. saprophyticus* (n* = 4/16)	–	–	25	0	0	6.2	0	6.2	–	–	25	12.5	–	–	25	0	–	–	25	0	75	62.5	0	0
*Acinetobacter* spp. (n* = 4/14)	50	35.7	100	64.2	25	7.1	25	14.2	25	7.1	50	14.2	–	–	75	78.5	25	7.1	–	–	–	–	–	–
*Proteus* spp. (n* = 4/19)	0	63.1	0	89.4	0	0	0	31.5	0	10.5	25	31.5	0	10.5	–	–	0	0	–	–	–	–	–	–
*K. oxytoca* (n* = 3/7)	0	28.5	0	100	0	42.8	0	57.1	0	42.8	0	28.5	0	28.5	0	0	0	42.8	–	–	–	–	–	–
*Citrobacter* spp. (n*=1/8)	0	100	0	87.5	0	25	0	12.5	0	12.5	0	12.5	0	12.5	0	12.5	0	25	–	–	–	–	–	–
*S. aureus* (n*=1/6)	0	33.3	–	–	–	–	–	–	–	–	–	–	–	–	–	–	–	–	0	0	100	83.3	0	16.6
*Enterobacter* spp. (n*=1/7)	0	71.4	0	71.4	0	14.2	0	14.2	0	14.2	–	–	0	0	0	28.5	0	14.2	–	–	–	–	–	–

n* inpatient/outpatient, *IN* inpatient, *OUT* outpatient, *AMX* amoxicillin, *CZO* cefazolin, *CRO* ceftriaxone, *NAL* nalidixic acid, *NOR* norfloxacin, *SXT* cotrimoxazole, *GEN* gentamicin, *NIT* nitrofurantoin, *CTX* cefotaxime, *VAN* vancomycin, *PEN* penicillin, *ERY* erythromycin

Other Gram Negative organisms isolated (data not shown as a figure) were least resistant to gentamycin (Out 13.4, In 50) followed by norfloxacin (Out 21.4, In 24.9) and cefotaxime (Out 19.8, In 37). *Pseudomonas* spp. was excluded from the data analysis in this study since there was only 1.4 % incidence and it is usually considered to be nosocomial infections. Gram positives on the other hand were sensitive to most of the antimicrobials with 100 % sensitive to vancomycin followed by nitrofurantoin (95.1 %) respectively. The resistance rates for third generation cephalosporin (which is a surrogate marker of ESBL) is striking (CRO 15.8 %, CTX 15.9 %). Since there is no particular method used to define ESBL in this study there is a need for further studies required.

## Discussion

Urinary tract infection is one of the most common and serious infectious disease which affects all age groups of people including men, women and children worldwide [1, 11]. Empirical treatment should be based on local data regarding common uropathogens and their antimicrobial susceptibility to available antibiotics [11, 12]. This is the first study conducted to determine the prevalence of UTI, the effect of gender and age on its frequency and the susceptibility profile of uropathogens isolated in patients visiting JDW/National Referral Hospital. This study provides valuable laboratory data to monitor the status of antimicrobial resistance among uropathogens and to improve empirical treatment recommendations in a specific region.

In this study a total of 6030 patient’s urine samples were analyzed, only 14.1 % had a bacterial UTI and 0.14 % had *Candida* spp. infection. This finding has been similar in line with studies done in Iran by Farajnia et al. [4] which have an isolation rate of 13.2 %. However the isolation rate of bacteria from urine is comparatively lower than the reports from other parts of the world [3, 14]. This might have been due to limited documentation of clinical syndrome, previous therapeutic details and sample provision as ascertaining definite hospital acquired UTI was difficult. Free primary medical service provided in the country and irrational use of laboratory service or the studies might have been based on the retrospective surveillance. The other factors includes the discrepancies depending on the sample collection and processing performed by different staff on duties at different time despite the establishment of CLSI method in the microbiology lab.

The incidence of infection was higher among females of sub group 18–26 years in the current study. Similar reports were observed by Dash et al. [3] in India and Akoachere et al. in Cameroonian town [14]. In our present study *E. coli* accounted 79.3 % followed by *K. pneumoniae* 4.1 % to be the main etiologic agent causing urinary tract infection. *Escherichia coli* being the pre-dominated cause, this correlates exactly with the other studies conducted by Schmiemann et al. in Germany [15] which also accounted *E. coli* to be the causative agent with 79 % prevalence. Similar studies conducted in developing countries [5, 7, 9, 14], proved *E. coli* to be the causative agent followed by *K. pneumoniae*.

Most of the international guidelines for treatment of community acquired urinary tract infection suggest co-trimoxazole, amoxycillin/ampicillin, norfloxacin as a preferred empirical treatment [11]. However the guidelines have a suggestion that local antimicrobial susceptibility pattern must be taken in account before choosing any antibiotics [12]. And also the rate of >20 % resistance in the community is the most accepted rate for empiric decisions as suggested by the infectious diseases society of America. All the studies conducted in various part of the world have proved the resistance of uropathogens to commonly prescribed antimicrobial agents such as amoxycillin/ampicillin and co-trimoxazole [3, 6, 7, 9–11, 13–16]. The present study also supports that resistance to these antimicrobials have been evolved with the resistance rate of 80.3, 71.4 and 58.4 % respectively for nalidixic acid, amoxycillin and co-trimoxazole. This preliminary finding of high resistance rate for the commonly used antibiotics has a huge public health implications. Currently health care is free in the country including consultations, laboratory investigations and treatment. This high resistance might warrant the change of empirical antibiotic treatment of UTI in the country resulting into the change of the national essential drug list and existing guidelines. In addition this high resistance rate is surprising in the country where over the counter antibiotics prescribing is almost non-existed.

## Conclusion

This study findings showed that *E. coli* was the pre dominant uropathogen with overall 79.3 % (outpatient 81.1, inpatient 69.5) prevalence and its antibiotic susceptibility should be considered for treating community acquired urinary tract infection empirically. Since the rate of >20 % resistance in the community is the most accepted rate for empiric decision as suggested by infectious diseases society of America(IDSA), nitrofurantoin remains the choice for treatment of community acquired urinary tract infection empirically. The antibiotic resistance among the uropathogens is an evolving process so a routine surveillance to monitor the etiologic agents of UTI and the resistance pattern should be carried out timely to choose the most effective empirical treatment by the physicians. The third generation cephalosporin resistance (which is a surrogate marker of ESBL) was 16.1 % in outpatient and 16.7 % approximately in inpatient setting which is alarmingly high and there is need for further studies.
